# Body mass index–based predictions and personalized clinical strategies for colorectal cancer in the context of PPPM

**DOI:** 10.1007/s13167-022-00306-0

**Published:** 2022-12-02

**Authors:** Yun-Jia Gu, Li-Ming Chen, Mu-En Gu, Hong-Xiao Xu, Jing Li, Lu-Yi Wu

**Affiliations:** 1 Yueyang Hospital of Integrated Chinese and Western Medicine, Shanghai University of Traditional Chinese Medicine, No.110 Ganhe Road, Shanghai, 200437 China; 2 Shanghai Qigong Research Institute, Shanghai University of Traditional Chinese Medicine, No. 650 South Wanping Road, Shanghai, 200030 China

**Keywords:** Colorectal cancer, Obesity, BMI (body mass index), SMI (skeletal muscle index), ERAS (enhanced recovery after surgery), Weight management, Predictive, preventive and personalized medicine (3PM/PPPM)

## Abstract

Currently colorectal cancer (CRC) is the third most prevalent cancer worldwide. Body mass index (BMI) is frequently used in CRC screening and risk assessment to quantitatively evaluate weight. However, the impact of BMI on clinical strategies for CRC has received little attention. Within the framework of the predictive, preventive, and personalized medicine (3PM/PPPM), we hypothesized that BMI stratification would affect the primary, secondary, and tertiary care options for CRC and we conducted a critical evidence-based review. BMI dynamically influences CRC outcomes, which helps avoiding adverse treatment effects. The outcome of surgical and radiation treatment is adversely affected by overweight (BMI ≥ 30) or underweight (BMI < 20). A number of interventions, such as enhanced recovery after surgery and robotic surgery, can be applied to CRC at all levels of BMI. BMI-controlling modalities such as exercise, diet control, nutritional therapy, and medications may be potentially beneficial for patients with CRC. Patients with overweight are advised to lose weight through diet, medication, and physical activity while patients suffering of underweight require more focus on nutrition. BMI assists patients with CRC in better managing their weight, which decreases the incidence of adverse prognostic events during treatment. BMI is accessible, noninvasive, and highly predictive of clinical outcomes in CRC. The cost–benefit of the PPPM paradigm in developing countries can be advanced, and the clinical benefit for patients can be improved with the promotion of BMI-based clinical strategy models for CRC.

## Objectives for the review

The significance of body mass index (BMI) in colorectal cancer (CRC) preventative screening has been well established in previous studies that focused on the effects of overweight (high BMI) and the screening and assessment of CRC risk using BMI [1–3]. Mainstream therapies such as radical surgery [4] and neoadjuvant chemotherapy [5] have been demonstrated to correlate with BMI. BMI may predict the outcome of treatment for CRC. However, a comprehensive review of these studies was not available to a personalized BMI-based clinical strategy for CRC. This paper discusses the role of BMI in the clinical management of CRC from the perspective of clinical treatment and weight management, thus promoting clinical strategies for CRC in the predictive, preventive, and personalized medicine (3PM/PPPM) paradigm.

## State of the art

### CRC, BMI, and PPPM

CRC is the third most diagnosed cancer worldwide and the second leading cause of cancer-related mortality [6]. Overweight is a high-risk factor for CRC and a major cause of death [7, 8]. The pathogenesis is mostly influenced by visceral abdominal fat and insulin storage [9]. As a simple means to quantify obesity by calculating height and weight, BMI is frequently used to screen for CRC morbidity and mortality [10]. It can be easily calculated by dividing the weight (kg) by the square of height (m). The application of BMI neither includes medical testing procedures, breaches patient privacy, nor endangers the patient’s body in any way. To meet the need for more multidimensional healthcare, the PPPM paradigm is widely used in the diagnosis and treatment of cancer [11, 12]. Under standardized medicine, BMI is often used only as a general stratification factor. PPPM considers BMI as an important node in multiple predictors, incorporating it into a wide range of diseases and health models, including cancer prediction [13].

### BMI in CRC primary care: a proven marker

Obesity is a well-recognized risk factor for CRC, which has been confirmed by multiple epidemiological evidence [14]. Most studies suggest that obesity (high BMI) has a role in the emergence of CRC [15]. **Morbidly obese individuals (BMI ≥ 30 kg/m**^**2**^**) have a higher risk of developing CRC (risk ratio [RR] = 1.93; 95% confidence interval [CI], 1.15–3.25)**[6].**Males may be more at risk than females** [16]**,** **although maternal obesity (BMI ≥ 30 kg/m**^**2**^**) also increases the risk of CRC diagnosis in offspring (adjusted hazard ratio [aHR] = 2.51; 95% CI, 1.05–6.02)** [17]**.** Insulin resistance, hyperinsulinemia, elevated leptin levels, and the development of an inflammatory microenvironment can promote the growth and proliferation of colon cancer cells [9, 10, 18]. Therefore, obesity-associated CRC is considered a subtype of CRC. The addition of BMI as a proven marker in CRC screening programs worldwide has been scientifically recommended [19]. Longitudinal studies have established screening methods that combine BMI with exposure time [20], implying that weight control may be effective in CRC prevention. The focus of this paper is put on how BMI can be introduced into secondary and tertiary care for CRC, a more developmental topic compared to primary care. Bariatric surgery, dietary modification, calorie restriction, and other types of weight control have the potential to reduce the risk of CRC. This will be reviewed in detail in the following section.

### BMI in CRC secondary and tertiary care: ready for action

Individuals diagnosed with CRC are often offered standardized treatment based on guidelines aimed at preventing metastases, complications, and death, i.e., secondary and tertiary care for CRC. The PPPM paradigm seeks a higher quality of care, using more systems medicine, anthropometric, and even multicultural tools to define patients more precisely, with BMI being an important and fundamental factor. Excessive high or low BMI poses a significant impact on the prognosis and survival of patients with CRC receiving clinical treatment. **High BMI is a determinant in the progression of CRC** [21]**, while a low BMI is associated with an increased risk of death, a poorer prognosis, and malnutrition in patients with CRC** [22, 23]. **Notably, patients suffering from CRC with low or consistently declining BMI may be more susceptible to side effects or even death due to nutritional deficiencies or muscle loss** [24, 25]**.** Weight management after diagnosis also affects treatment outcomes. Currently, the mainstream therapies for treating CRC include laparoscopy treatment, surgery, adjuvant chemotherapy, systemic therapy, and radiotherapy [26–28]. In the following paragraphs, we will discuss treatment modalities and weight management to investigate the impact of BMI on CRC receiving appropriate treatment.

#### BMI-based predictions in surgical treatment of CRC

##### Surgical treatment—open surgery and laproscopy

Open surgery and laparoscopic surgery are the two main categories for the surgical treatment of CRC. Usually, laparoscopic surgery is used to treat locally advanced, non-metastasis colon cancer that is resectable. Since the 1990s, it has been widely used due to its exceptional safety and efficacy [29]. **For patients with stage II/III colon cancer, laparoscopic surgery is a reasonable selection, while patients with BMI ≥ 30 kg/m**^**2**^ **may need to carefully consider the surgical procedure** [30]**. As BMI increases, patients are more likely to switch from laparoscopic surgery to open surgery, with a subsequent increase in the incidence of surgical complications** [31]**.** Patients with obesity have higher technical requirements for laparoscopic surgery than those without obesity [32]. Due to the thickening of the mesentery, laparoscopic surgery may result in vessel dissection and difficulty in ligation. Obesity also contributes to postoperative infections, which cause postoperative wound complications due to inadequate concentration of antibiotics in the tissues [33]. **A pooled analysis of comparative studies also suggested that patients of CRC with a high BMI (≥ 30 kg/m**^**2**^**) may have poor perioperative outcomes, such as longer operative times and greater blood loss** [34]**.** However, there is no difference in oncological outcomes between open surgery and laparoscopic surgery [35, 36]. Patients with obesity and high BMI need to carefully select for open surgical treatment options and laparoscopic surgery treatment. Furthermore, their postoperative recovery should be closely observed to take active care of the surgical wound, thus minimizing the adverse effects of postoperative complications.

##### Surgical treatment—emergency open surgery

Emergency open surgery is routinely performed in patients with surgical emergencies such as intestinal obstruction or perforation in CRC [37]. Since the obstruction of CRC often emerges in a more advanced stage of liver metastasis, and perforation usually develops in near tumors [38], the implementation of emergency surgery is inherently risky. **Surgery-related stoma problems are common in patients with CRC with left-sided intestinal obstruction and high BMI (BMI ≥ 30.0 kg/m**^**2**^**), which is not conducive to prognostic care** [39]**.** This result demonstrates that patients with CRC need more attention to their prognosis and better postoperative management after emergency surgery.

##### Surgical treatment—colostomy

Colostomy can also be employed in treating left-sided obstructive colon cancer, **whereas patients with high BMI are at increased risk of parastomal hernias and early skin irritation** [40]**.** If this treatment is chosen, special attention should be paid to post-operative recovery and prognosis for patients with high BMI.

##### Surgical treatment—robotic-assisted surgery

Robotic-assisted surgery is often used as a minimally invasive procedure for cases of colon cancer requiring total mesenteric resection. This kind of surgery is safer than traditional open surgery and as effective as laparoscopic surgery [41]. **It is equally applicable to patients with BMI in various classification** [42]. However, patients need to know that robotic surgery is more costly and requires a longer time to perform [43].

##### Surgical treatment—enhanced recovery after surgery

Enhanced recovery after surgery (ERAS) is a comprehensive intervention program in the perioperative period that reduces the stress of surgery and promotes rapid recovery through multidisciplinary collaboration between surgeons, anesthetists, and surgical nurses [44]. There is multiple evidence to support that ERAS can reduce the length of stay, reduce healthcare costs, and improve the quality of recovery in patients undergoing CRC surgery [45, 46]. There have been studies comparing the outcomes of laparoscopic CRC surgery with ERAS in patients with various BMI classifications and types of obesity. **However, no significant differences between obesity subgroups demonstrated the generalizability of ERAS for CRC surgery** [47]**.**

##### Low BMI as a risk factor in the surgical treatment of CRC

**In contrast, patients with CRC and excessively low BMI are more likely to die after surgical treatment due to impaired nutrition and immune deficiency** [4]**. Patients with low BMI (15–19.99 kg/m**^**2**^**) also have worse long-term survival than those with a normal BMI** [48]**.** Therefore, nutritional support can be considered to improve immunity before surgical treatment, and the rehabilitation of the patient should be closely monitored after surgery.

##### Hepatic surgery for metastatic CRC

Hepatectomy is the most effective treatment option to cure or prolong the life of patients with CRC liver metastases. **Patients with a BMI ≥ 28 kg/m**^**2**^ **are more likely to develop steatohepatitis because of steatosis** [49]**. Minimally invasive liver surgery is a preferable alternative for individuals with obesity and a higher BMI due to its increased safety and effectiveness** compared to standard open resection or laparoscopic resection [50]. Skeletal muscle loss (SMI) can be used to predict complications because BMI does not accurately predict surgical complications following hepatectomy [51].

##### Cytoreductive surgery with hyperthermic intraoperative intraperitoneal chemotherapy

CRC is usually followed by peritoneal metastases and progresses to peritoneal carcinoma in certain cases [52]. Tumor cell reduction combined with intraoperative heat-infused intraperitoneal chemotherapy (CRS + HIPEC) is currently an efficient treatment for this disease. CRS is a surgical procedure that employs argon knife cautery to remove visible tumors from the abdominal cavity as completely as possible. HIPEC is a regional treatment of abdominal tumors combining abdominal heat and chemotherapy, which has enhanced pharmacokinetic advantages over conventional systemic therapy. In this treatment, an infusion heated to a certain temperature is mixed with chemotherapeutic agents and instilled into the abdominal cavity. **There was no significant difference in the efficacy of this treatment between patients with high BMI and those with BMI, suggesting that it is suitable for both types of patients** [53]**.**

#### BMI-based predictions in adjuvant chemotherapy of CRC

##### High BMI in adjuvant chemotherapy of CRC

Adjuvant chemotherapy is frequently recommended for the recovery treatment of CRC up to 3 months following surgery. Fluorouracil-based single-agent regimens, such as oral capecitabine and folinic acid-modulated 5-fluorouracil regimen (5-FU), are among the clinically applicable adjuvant chemotherapy regimens and are among the clinically applicable adjuvant chemotherapy regimens. Oxaliplatin plus capecitabine (CapeOx regimen), oxaliplatin plus 5-FU plus leucovorin (mFOLFOX6) regimen, and 5-fluorouracil and levamisole (5FU-LEV) regimen are a few examples of different combination chemotherapy regimens. **Adverse prognosis events after adjuvant chemotherapy, such as relapse and death, are more likely to occur in patients with high BMI (≥ 30 kg/m**^**2**^**)** [54, 55]**. More specifically, patients with a higher BMI may be more susceptible to the adverse effects of adjuvant chemotherapy, such as nausea, vomiting, and peripheral neuropathy** [56], while taking 5-FU-based chemotherapy for metastatic CRC (mCRC). **Male patients with extreme obesity had a 16% higher mortality rate than patients of normal weight, and they may have a worse prognosis than female patients,** in particular, due to increased buildup of abdominal or central fat [57]. **Those with a BMI > 35.0 kg/m**^**2**^ **demonstrated a considerably increased risk of CRC recurrence and even mortality following combination chemotherapy with 5-FU, 5-FU plus leucovorin/levamisole regimens** [58]**.** Patients with a greater BMI who received the drugs 5-FU and oxaliplatin had a higher rate of cessation, depression symptoms, and less social support [59]. A higher BMI was linked to peripheral neuropathy in patients with CRC receiving the oxaliplatin regimen combined with chemotherapy [60].

##### Low BMI in adjuvant chemotherapy of CRC

**A relapse following adjuvant chemotherapy is more common in patients with abnormally low BMI (< 20 kg/m**^**2**^**). Furthermore, compared to patients with normal BMI, they have a considerably shorter disease-free survival rate** [57]**.** Acute pancreatitis is a rare consequence of CRC that can also occur in underweight patients [61]. Therefore, adjuvant chemotherapy regimens should be carefully planned for patients with abnormally high or low BMI. The adjuvant chemotherapy prognosis and side effects should also be carefully considered.

##### Systematic therapy and bevacizumab for metastatic CRC

The standard treatment for initially unresectable liver is mCRC hepatectomy. However, more patients are choosing systemic therapy instead [62]. The commonly used systemic regimens in clinical practice include mFOLFOX6 plus bevacizumab/cetuximab, CapeOx plus bevacizumab, FOLFIRI (5-fluorouracil, leucovorin, and irinotecan), and FOLFOXIRI (folinic acid, 5-fluorouracil, oxaliplatin and irinotecan). **A previous study showed that mCRC patients with high BMI were more likely to develop steatosis and liver injury after liver resection following systemic therapy (including 5-FU, oxaliplatin, irinotecan, and the FOLFOXIRI regimen)** [63]**.** Additionally, patients with mCRC may experience a decrease in BMI after systemic therapy, accompanied by the development of ongoing decreased SMI. The decrease may be caused by the cachexia of the advanced disease, and it cannot demonstrate that the decrease in BMI is beneficial to the prognosis of mCRC [64–66]. Patients with obesity should have their BMI constantly monitored, and a drop in BMI should be considered a predictor of disease regression.

Bevacizumab is the primarily targeted therapy for mCRC that inhibits tumor angiogenesis by blocking the EGFR/VEGF receptor pathway. Compared with patients with normal BMI, **patients with BMI ≥ 25 kg/m**^**2**^ **may have a shorter tumor progression time because visceral fat can induce the accumulation of tumor factors** [67]**.** Among patients receiving chemotherapy and bevacizumab-targeted therapy, **those with excessively low or high BMI had a significantly shorter overall survival than normal individuals **[68]**.** However, some research has concluded that BMI is not a predictor of prognosis for the addition of targeted therapy to chemotherapy [69]. More evidence needs to be analyzed to determine the relationship between CRC-targeted therapy and BMI.

#### Treatment for rectal cancer

Surgery, adjuvant chemotherapy, systemic therapy, and simultaneous radiotherapy are all used to treat rectal cancer. Similar to colon cancer, a higher BMI increases the risk of rectal cancer surgery and the operation difficulty [70, 71]. Moreover, continuous growth in BMI is associated with higher rates of post-operative complications and longer post-operative hospital stays [72, 73]. **Patients with excessively low BMI (< 18.5 kg/m**^**2**^**) have a poorer early and long-term prognosis after rectal cancer surgery** [74]**,** leading to a higher risk of acute organ toxicity and even death [25].

##### Abdominoperineal resection

**Abdominoperineal resection is a specific surgical treatment for rectal cancer, especially for patients with obesity and a high BMI. Male patients with obesity and rectal cancer are more likely than normal-weight male and female patients to experience a local recurrence.** They might be unable to retain their sphincter due to pelvic stenosis and increasing obesity [75]. Furthermore, the overall survival rate of patients with obesity who undergo this procedure is lower [76]. Based on these results, the postoperative rehabilitation and prognosis of male patients with high BMI need to be closely observed.

##### Simultaneous radiotherapy

Simultaneous radiotherapy is the standard treatment for inoperable locally advanced rectal cancer. Commonly used treatment regimens are radiotherapy plus capecitabine or radiotherapy plus continuous infusion of 5-FU. Male patients with obesity and high BMI have more adipose tissue in the prostate-rectum junction, and the dose of radiotherapy to the rectal wall is compromised. Therefore, individualized radiotherapy protocols may be more suitable for these patients, such as increasing radiotherapy dose or intensifying systemic therapy [25].

However, **extra attention is needed if a consistent trend of decreasing BMI is observed. The toxicity of radiotherapy may affect treatment efficacy and cause diarrhea, renal insufficiency, and radiation proctitis, leading to malnutrition in patients with a BMI loss ≥ 7%** [22]**.** Compared to normal patients, patients with rectal cancer who has an excessively low BMI have a significantly lower overall survival rate [77, 78]. Apart from BMI changes, these patients may receive individualized dietary advice and manual nutritional support to evaluate spontaneous food intake, toxicity tolerance, and nutritional status [79]. The impact of BMI on other treatment options, such as systemic therapy and adjuvant chemotherapy, can be reviewed in the previous section on colon cancer.

### BMI management strategy of CRC: comprehensive long-term efforts

According to Hu et al. [23], patients with CRC should avoid excessive weight loss and weight gain. Excessively high or low BMI seriously impacts the outcome and prognosis of CRC. Therefore, a fat reduction strategy should be formulated for patients with CRC and excessively high BMI ( ≥ 35 kg/m^2^) [80]. **For patients with excessively low BMI (< 18.5 kg/m**^**2**^**), supplemental nutritional intake can be considered to increase their body weight [**79**], thus increasing their BMI to a more reasonable value.** This part will focus on patients with CRC and excessively high or low BMI. Based on previous findings, a detailed discussion is provided to ameliorate BMI in patients with CRC by combining the current popular weight management methods (e.g., bariatric surgery, diet control, physical activity, and obesity pharmacotherapies).

#### BMI management approaches for CRC with excessively high BMI

##### Non-pharmacological therapy—bariatric surgery

As a long-term weight loss solution, bariatric surgery (BRS) can help with many obesity-related comorbidities. The common procedures are gastric bypass, gastric banding, or sleeve gastrectomy [81]. The role of BRS for CRC is focused on primary care. Practitioners aim to reduce the risk of various diseases, including CRC, by performing BRS on overweight individuals (mainly those with a BMI ≥ 35kg/m^2^). However, there are conflicting findings as to whether BRS reduces the risk of CRC. High-level evidence suggests that patients with obesity undergoing BRS had over 30% lower risk of colorectal cancer compared to patients with obesity not receiving BRS [82, 83]. BRS has a long-term and durable protective effect on CRC patients with high BMI. It can effectively prevent the development of CRC lesions and reduce postoperative complications through weight loss, facilitating the postoperative prognosis [84, 85]. Other studies have shown that BRS not only fails to reduce [86] but increases the short-term risk of CRC in populations under 50 years of age [87], particularly in the case of gastric bypass surgery [81]. This may be attributed to the inflammatory environment following BRS stimulating hyperproliferation of the intestinal mucosa. The treatment guidelines for CRC should be followed to improve the screening for various risk factors, such as age, sex, tumor stage, and the presence of metastases obstruction symptoms [88]. Based on current findings, BRS is an approach to prevent overweight-related diseases, but the impact on CRC risk remains inconclusive. For the time being, there is no evidence of benefit from BRS after CRC diagnosis. BRS is not recommended temporarily in the CRC population due to concerns about the effect on digestive tract function itself.

##### Non-pharmacological therapy—diet control

Overweight and obesity result from an imbalance between energy intake and expenditure. A case–control study also demonstrated that patients with CRC have a significantly higher total calorie intake than the normal population, leading to a high BMI in these patients [89]. Patients with CRC and higher BMI tend to favor an unbalanced diet of high fat and lack of fruits, vegetables, and dietary fiber. This unhealthy diet affects the visceral adipose tissue (VAT) profile and leads to altered metabolic pathways, promoting the development of CRC pathologies [90, 91]. Additionally, refined grains, alcohol, processed meats, and red meats are detrimental to the prognosis of patients with CRC [92]. Therefore, patients with high BMI require comprehensive dietary modification. **A single-arm exploratory study conducted a diet-mediated weight loss intervention in 20 adults with high BMI. The results revealed that weight loss achieved through a low-energy dietary replacement diet could effectively reduce tumor-related markers in colon tissue and serum** [93]**.** Dietary fibers can be supplemented by eating fruits and vegetables (e.g., onions, apples, berries, cucumbers, sweet potatoes, tomatoes, and peppers); carbohydrate can be supplemented with unrefined grains and legumes; milk and dairy products can provide vitamin B12, lectins, and calcium; and protein can be obtained from poultry, fish, and other low-fat meats [88, 89]. The lectins provided by legumes may have an adjuvant effect on CRC [94].

##### Non-pharmacological therapy—chronic caloric restriction

Chronic caloric restriction (CR) has been the major dietary intervention to reduce weight and prevent cancer. As an alternative to CR, intermittent fasting (IF) has become a major research focus in recent years, which is more frequently used in basic research such as animal studies [95–97]. Nevertheless, the use of IF in the clinical treatment of CRC is poorly studied. No data are available on the positive impact of IF without weight loss and the impact of changes in diet quality and physical activity patterns on the prognostic outcome of CRC [98]. It has been suggested that prolonged perioperative fasting in patients with CRC may lead to longer hospital stays [99]. The above studies suggest that IF can be used as an effective tool for patients with obesity and high BMI to reduce body fat and thus prevent CRC. Currently, IF is not recommended for patients with CRC and higher BMI under treatment. When IF was used before CRC diagnosis, it should not be continued during treatment, except as part of a clinical trial. Physicians should explain the risks and benefits of IF to patients if they wish to receive it during a long-term treatment [98].

##### Non-pharmacological therapy—physical activity

**Current research indicates that a moderate participant in physical activity (PA) has a protective effect on patients with CRC and high BMI and may improve the prognosis of these patients** [100]**.** Regular and moderate physical activity increases basal metabolism and improves tissue oxygenation. As a result, metabolic efficiency and capacity can be facilitated, ultimately reducing the volume of body fat and adipose tissue and mitigating the impact of obesity on patients with CRC [101]. PA reduces circulating insulin and inflammatory markers in CRC [102, 103] thus improving the prognosis of patients. However, some studies have also shown that PA does not ameliorate HRQoL (health-related quality of life) and fatigue after CRC surgery [104]. Short-term (two-week) home-based physical activity after surgery also has no effect on self-assessed physical recovery [105]. Lee et al. [106] noted that a six-week home-based, supervised, and mixed exercise intervention could improve physical activity levels and fitness in survivors of stage II to III CRC. Patients with CRC may suffer from chronic fatigue and cardiac function problems that limit the conduct of PA [107]. Therefore, a more detailed long-term PA schedule should be established to optimize the amount, type, and intensity of exercise. Ultimately, exercise guidelines that better meet the needs of patients can be developed, alleviating the impact of high BMI on the treatment received by patients with CRC.

##### Pharmacological interventions in BMI management

Based on the nature, action mode, efficacy, and side effects of obesity drugs, they can be classified into short-term and long-term anti-obesity drugs [108]. Short-term anti-obesity drugs include amphetamine, diethylpropion, and benzphetamine; long-term anti-obesity drugs include orlistat, lorcaserin, and liraglutide. Some anti-obesity drugs are addictive and easily abused, with various side effects such as dizziness, heart palpitations, and gastrointestinal adverse reactions. When patients with high BMI take anti-obesity drugs, the associated contraindications need to be confirmed, and the drug dose should be strictly controlled. In addition to these drugs, **metformin** [109] **and berberine** [110] can also affect weight reduction and have anti-tumor activity.

##### Metformin

Metformin is an oral hypoglycemic drug and an adjunct to the treatment of obesity. It lowers blood glucose in patients with obesity by reducing hepatic glucose production and stimulating glucose uptake by peripheral tissues (muscle and fat) [111] and contributes to the weight loss [109, 112]. Current evidence suggests that metformin has an adjuvant effect on the treatment of cancer, particularly CRC. Metformin reduces the risk of CRC [113], reducing serum inflammatory factors[103], and inhibits tumor growth through the AMPK/mTOR pathway, mTOR/AKT pathway, and LKB1/AMPK pathway, thus facilitating the prognosis of patients [114–116]. Metformin improves the overall survival of patients with CRC receiving adjuvant chemotherapy and alleviates side effects such as chronic peripheral neuropathy caused by chemotherapy drugs [117–120]. It also enhances the efficacy of radiotherapy to promote tumor regression in patients with CRC [121]. Recent evidence suggests that metformin may function as a potential radiosensitizer in clinical antitumor therapy. However, its action and molecular mechanisms need to be further investigated.

Currently, only a few studies examined CRC populations with high BMI. **Miranda et al.** [122] **suggested that patients with CRC and a BMI ≥ 30 kg/m**^**2**^ **could significantly benefit from metformin combined with a 5-FU regimen.** Refined subgrouping of BMI is still needed to clarify the prognostic impact of metformin on patients with CRC and high BMI. Most of the evidence implies that the overall impact of metformin on patients with CRC and high BMI is positive. It has a multifaceted therapeutic effect through weight loss and adjuvant cancer treatment, which requires to be refined with further clinical evidence.

##### Berberine

Berberine is a natural isoquinoline alkaloid derived from the genus Berberis [123]. Current evidence suggests that berberine has a weight loss effect on patients with obesity [110]. In addition, it has anti-cancer effects and can target CRC tumors involved in proliferation, invasion, angiogenesis, and metastasis [124, 125]. Chen et al. found that berberine inhibited inflammatory cancer transformation of CRC by modulating mitofusin-2 (MFN2) to reduce colitis exacerbated by obesity [126]. Nevertheless, no relevant clinical studies on CRC populations with high BMI have been conducted. In conclusion, berberine is a promising drug for treating CRC. Further comprehensive investigations are necessary to elucidate the effect of berberine on patients with CRC and high BMI.

#### BMI management approaches for CRC with excessively low BMI

Special consideration should be taken to patients with CRC and low BMI (< 18.5 kg/m^2^). An excessively low BMI or a sustained decrease in BMI can negatively affect the survival and prognosis of patients with CRC [57, 68], resulting in the occurrence of weight and nutritional loss, producing cachexia[127].

##### Oral nutritional supplements

Oral nutritional supplements (ONS) are commonly used in clinical practice to supplement the nutrition not available from the normal diet in patients at risk of malnutrition [128]. Administration of ONS reduces the incidence of skeletal mucle loss and sarcopenia and improves chemotherapy tolerance in patients at nutritional risk (expected reduction in BMI and SMI) after CRC surgery [129]. In addition, supplementation of vitamin D and Omega-3 fatty acids in the daily diet counteract the nutrient deficiencies of patients with low BMI and decrease the inflammatory status after chemotherapy [130].

##### Other suggestions

In order to closely monitor the nutritional status of these patients, individualized nutritional interventions, nutritional counseling and education, and manual nutritional support such as regular dietary outpatient visits should also be performed which is well in agreement with PPPM strategies and attitude towards better healthcare [131–133]. If a persistent decline in BMI is observed, it is necessary to identify other risk factors to determine the cause of the decline in BMI, including the muscle loss or malignancy of the disease [65].

## Discussion on reviewed knowledge and data

This paper reviews the impact of BMI on the prognosis of various treatments for CRC, providing a comprehensive summary and evaluation of the current feasible methods for managing BMI in patients with CRC. The impact of different BMI levels on various treatments for CRC has been presented in Table 1 and Table 2. The current evidence indicates that BMI takes an important part in the progression and prognosis of the disease in patients with CRC.

**Table 1:**
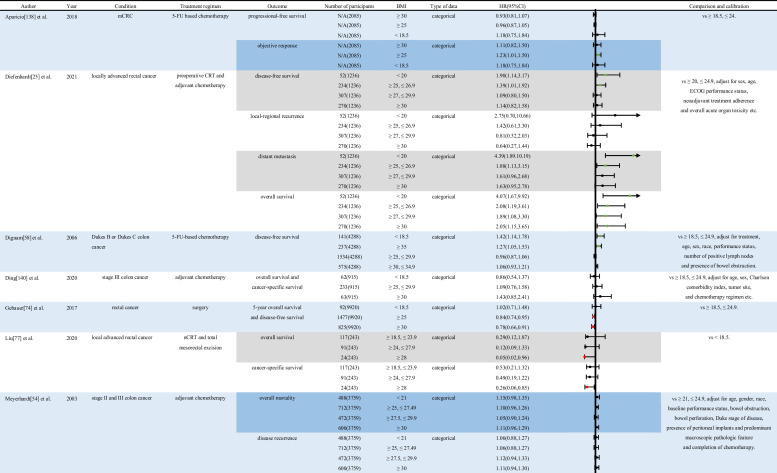

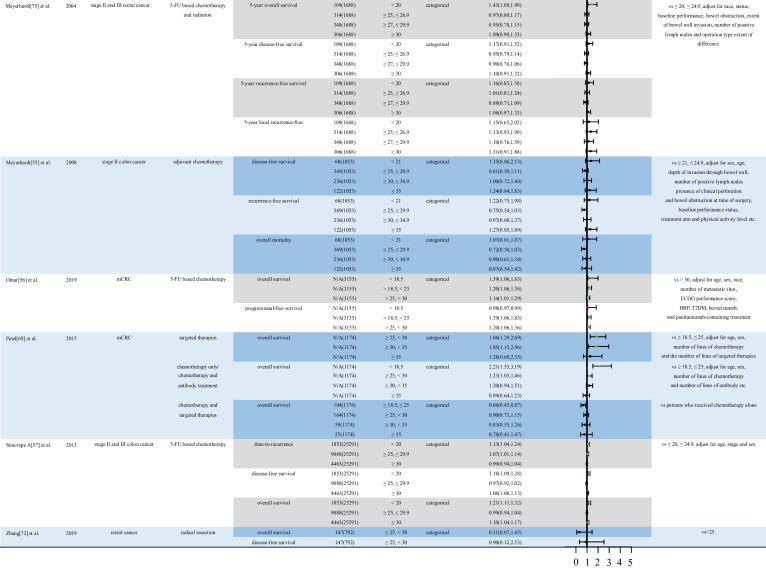
Impact of different BMI classifications on the prognostic risk of various treatments for CRC. Relevant studies on patients with CRC receiving different treatments were searched in the PubMed database to examine the impact of BMI on diverse prognostic outcomes. The diversity between different outcomes is shown using the RStudio software’s “Forest plot” package. The results of the analysis are shown in the table. Hazard ratios (HRs), treatment regimen, and associated 95% confidence interval (CIs) of outcomes (e.g., overall survival, disease-free survival, and cancer-specific survival) were extracted to evaluate the effect of BMI on CRC treatment statistically. Abbreviation: *5-FU*, 5-fluorouracil; *BMI*, body mass index; *N/A*, not available; *CI*, confidence interval; *CRC*, colorectal cancer; *CRT*, chemoradiotherapy; *ECOG*, ECOG score standard; *HBP*, high blood pressure; *HRs*, hazard ratios; *mCRC*, metastatic colorectal cancer; *nCRT*, neoadjuvant chemoradiotherapy; *T2DM*, diabetes mellitus type 2. Note: Green points indicate the protective effect of the disease. Red points indicate the risk for the disease

**Table 2:**
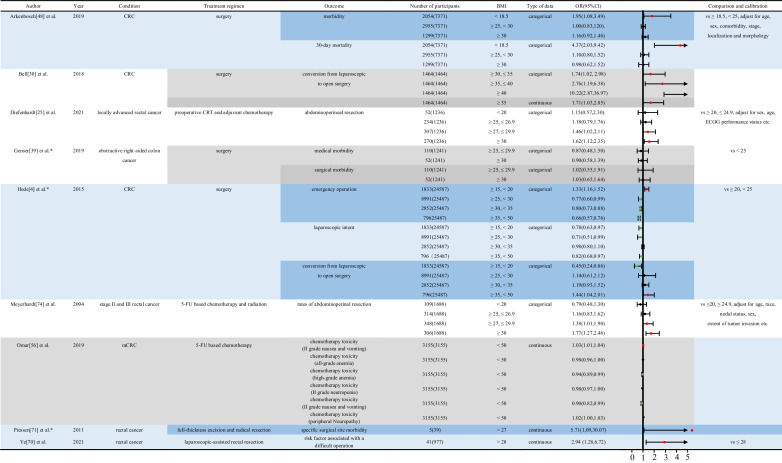
Impact of different classifications of BMI on short-term outcomes of various treatments for CRC. Relevant studies on patients with CRC receiving different treatments were searched in the PubMed database to examine the impact of BMI on short-term outcomes of CRC treatments. Relevant data are presented in tables. The “Forest plot” package of the RStudio software was used to show the diversity between the various outcomes. Abbreviation: *5-FU*, 5-fluorouracil; *BMI*, body mass index; *CI*, confidence interval; *N/A*, not available; *CRC*, colorectal cancer; *CRT*, chemoradiotherapy; *ECOG*, ECOG score standard; *HBP*, high blood pressure; *mCRC*, metastatic colorectal cancer; *nCRT*, neoadjuvant chemoradiotherapy; *ORs*, odd ratios; *T2DM*, diabetes mellitus type 2. *One-way logistic regression was used to evaluate the ORs for various BMI levels while determining treatment outcomes. The statistical program for social sciences 26.0 was used to extract and process data from the original manuscript. A two-way approach was used for all statistical analyses. Note: Green points indicate the protective effect of the disease. Red points indicate the risk for the disease

### BMI classification standard and ethnicity

According to the BMI classification by the World Health Organization (WHO), patients with underweight have a BMI < 18.5 kg/m^2^, patients with overweight range from 25.0 to 29.9 kg/m^2^, patients with obesity range from 30.0 to 35 kg/m^2^, patients with a BMI ≥ 35 kg/m^2^ are morbidly obese, and 18.5–24.9 kg/m^2^ is the most appropriate BMI range [134]. There are national and demographic differences in the BMI classification. Compared to European and American populations, there is a different association between BMI, body fat percentage, and health risks in Asian populations. The normal range of BMI for the Asian population is 18.5–22.9 kg/m^2^, and more than 25 kg/m^2^ is considered obese [135]. The ideal BMI range for Chinese people is 18.5–23.9 kg/m^2^ [136]. Clinicians should develop individualized weight management strategies based on the national and demographic characteristics of patients with CRC, thereby maintaining their BMI at the most reasonable level.

### Obesity paradox

Notably, the obesity paradox exists in CRC [15, 23]. The term *obesity paradox* first appeared in a US study about percutaneous coronary intervention (PCI) [137]. It refers to the trend of a U-shaped relationship between BMI and the corresponding symptoms of patients. Among patients with CRC, those with obesity and high BMI have poorer survival rates. **However, for patients with advanced CRC with a risk of malnutrition, maintaining a moderately high BMI (except for patients with morbid obesity and an excessive-high BMI) may be more helpful for their nutritional reserve and survival** [23]**. Aparicio et al. **[138] **also showed that patients with mCRC in the BMI range of 28–30 kg/m**^**2**^ **had a better prognosis than normal individuals.** A possible explanation is that patients with overweight have greater lean muscle mass than normal one, which helps improve the condition. In summary, the obesity paradox of CRC is more applicable to patients who are overweight and mildly obese. For patients who are morbidly obese, it is still recommended to better manage their BMI in combination with the fat reduction measures [139].

### Limitations of BMI and multi-factor assistance

BMI provides limited information and therefore relying on BMI alone is not sufficient. Based on the PPPM paradigm, a combination of factors such as gender, age, and ethnicity will help to better personalize healthcare. Some studies suggested that BMI is unrelated to the prognosis of CRC [140, 141]. The reason might be the delay in adjuvant chemotherapy and the specific combination chemotherapy of FOLFOXIRI plus bevacizumab and FOLFIRI/FOLFOX plus bevacizumab. This conclusion is not suitable for all patients with CRC. For women, abdominal obesity, high BMI at age 18, and subsequent weight gain are associated with a greater risk of CRC. While for men, weight gain in later years and overall obesity are important factors inducing CRC [2, 142]. Higher BMI is more likely to induce colorectal adenomas in black women [143].

Patients with CRC and low BMI (< 25 kg/m^2^) are susceptible to visceral obesity, which may have a negative impact on prognosis [144]. VAT and body composition on computed tomography (CT) should be combined with waist circumference (WC), waist-to-hip ratio, and other indicators for determining the degree of obesity. With these data, the authentic physical condition of patients can be comprehensively analyzed, allowing for appropriate clinical strategies [10].

An additional guiding direction for multifactorial assistance is to predict changes in BMI through simpler or more prospective representations. Models based on oculomics have been used to predict sarcopenia [145], which gives an idea for cross-sectional prediction. More usable predictive models based on genetic, multi-omic, and anthropometric associations may be formed in the future for PPPM.

### Potential BMI-based integrated management approaches

**Fig. 1:**
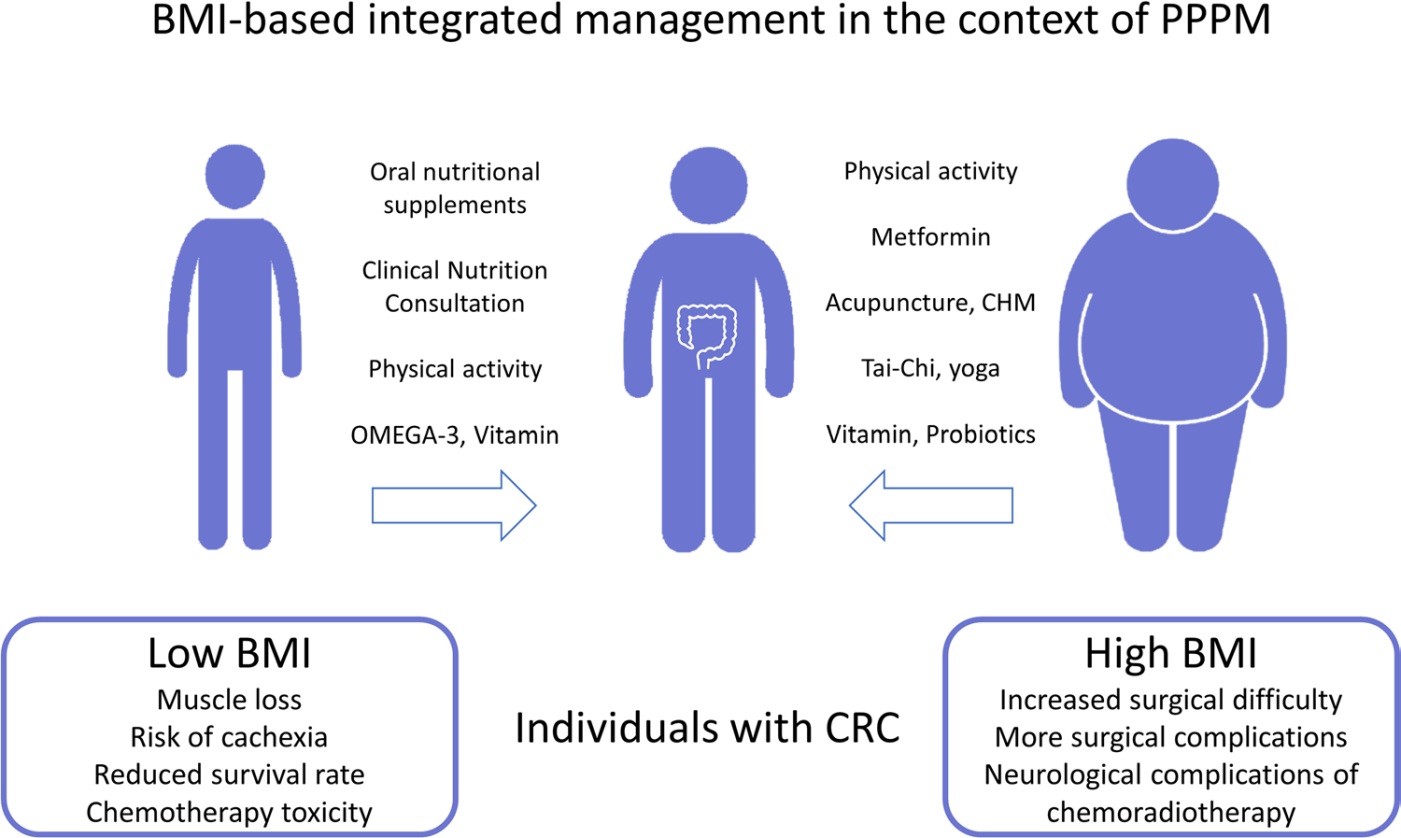
BMI-based integrated management in the context of PPPM. According to the culture and medical level of the patient, the multi-cancer and obesity domains are used as an extension of the evidence to develop a PPPM for patients with CRC based on BMI. Abbreviation: *BMI*, body mass index; *CRC*, colorectal cancer; *CHM*, Chinese herbal medicine; *PPPM*, predictive, preventive, and personalized medicine

From the evidence discussed above, we believe that BMI-based PPPM practice should be used throughout the full cycle of CRC care, from screening to postoperative recovery and systemic therapy (Fig. 1). It is possible to provide cost–benefit, which has been identified in the context of CRC screening [146]. Due to the limited evidence for the CRC population, practitioners and patients are advised to refer to the experience of BMI management in multi-cancer as well as chronic disease. The environmental applicability of PPPM practices for CRC would be considerably increased. We also consider multicultural factors to develop comprehensive programs to achieve the goal of personalized medicine. In full cycle CRC-BMI management, various types of complementary therapies with low side-effects, such as acupuncture [147, 148], Tai-Chi [149, 150], yoga [151, 152], Chinese herbal medicine [153, 154], and probiotics [155, 156], are recommended depending on the culture as well as the medical setting. Omega-3 fatty acids [157] are well evidenced and have potential to prevent cachexia. The vitamins [158–160] might also increase the survival rate. These factors align with PPPM advocacy [161]. The combination of physical activity and personalized nutrition should also receive attention, thus leading to the muscle gain rather than skeletal muscle loss which can be expected if no PA is exerted while. A well-balanced diet is given to patients with CRC [162]. Patients with CRC see a slight increase in BMI a few months following treatment (typically 6–24 months), a sign that tissue and functional status have recovered [163]. However, the recovery is subject to limited conditions, in which the BMI of patients should not be too high and there should be a limited intake of nutrients leading to poor tolerance of the treatment [23, 164]. In order to better determine the prognosis and recovery of patients, the BMI of patients with CRC should be regularly and continuously monitored.

## Conclusions and recommendations

BMI employs a straightforward formula that eliminates the need for invasive testing and improves the process of developing treatment strategies for patients with CRC. No biomarker can offer as simple and practical a measurement as BMI. In prior studies, BMI has been demonstrated to be useful in CRC primary care as a predictor consistent with the PPPM paradigm [13]. In this paper, we review the evidence for BMI in CRC secondary and tertiary care. We conclude that BMI can be considered as a full-cycle factor in the practice of PPPM for CRC. We provide Table 3 as a summary of the full text for quick overview and reference. BMI plays a powerful informative role in the development of personalized medicine protocols to the prevention of cancer cachexia. BMI surveillance reveal a poor prognosis and predicts progressive changes, which helps to avoid the risk of different therapies. From the PPPM perspective, BMI helps patients manage their weight and improve prognosis, allowing clinicians to design better personalized clinical strategies. The BMI-based PPPM paradigm is more effective and can be useful in a wide range of developing regions; moreover, it means that more patients can benefit from the PPPM. We recommend for further PPPM development and practical application, to include BMI as a monitoring factor in CRC care in wider range of regions.

**Table 3:** Full cycle BMI-based CRC management recommendations in the context of PPPM. Note: ↑with BMI increasing; ↓with BMI decreasing;—not BMI-related. Abbreviation: *5-FU*, 5-fluorouracil; *BMI*, body mass index; *CRC*, colorectal cancer; *HIPEC*, heat-infused intraperitoneal chemotherapy; *mCRC*, metastatic colorectal cancer

Clinical status/treatment	BMI	Suggested actions/risk prediction	Reference
Primary care			
Obese class III male	≥ 30	Regular CRC screening	[6, 16]
Maternal obesity	≥ 30	CRC risk screening for offspring	[17]
Bariatric surgery	≥ 30	Considered to reduce risk of overweight-related disease, but inconclusive for CRC	[82–87]
Individuals with elevated CRC markers	≥ 30	Meal replacement diet	[93]
Surgical treatment			
Stage II/III CRC	≥ 30	Preference for laparoscopic surgery	[30]
CRC laparoscopic surgery	↑	Being aware of the difficulties and complications	[31]
Antibiotic administration after CRC surgery	43 ± 10	Pay attention to the concentration of antibiotics to avoid wound infection	[33]
CRC perioperative period	≥ 25	Take greater care of surgical care to reduce the duration of the procedure and the amount of bleeding	[4, 34]
Emergency open surgery for left-sided intestinal obstruction	≥ 30	Prone to surgery-related complications	[39]
Colostomy	> 25	Increased risk of para-anastomotic hernia and early skin irritation	[40]
Robotic-assisted surgery for CRC	-	Suitable for patients at all classes of BMI	[42]
Enhanced recovery after surgery for CRC	-	Suitable for patients at all classes of BMI	[47]
After CRC surgery	< 20	Short and long-term mortality rates are higher than in normal weight patients	[4, 48]
Hepatectomy for mCRC	≥ 28	Higher risk of steatohepatitis and related complications	[49]
Minimally invasive liver surgery for mCRC	19.2–44.8	Safety profile for patients with high BMI and may be indicated	[50]
CRS + HIPEC	-	Suitable for patients at all classes of BMI	[53]
Adjuvant chemotherapy			
Adjuvant chemotherapy	≥ 30	Higher risk of experiencing adverse outcomes, such as relapse and death	[54, 55, 58, 59]
5-FU-based chemotherapy for mCRC	↑	Higher risk of chemotherapy side effects such as nausea and vomiting and peripheral neuropathy	[56]
5-FU-based chemotherapy for mCRC	↓	Higher risk of hematological toxicities (anemia and neutropenia)	[56]
Stage II–III, resected CRC, chemotherapy with 5-FU in monotherapy or associated with oxaliplatin	≥ 30	Higher risk of depression. Higher risk of treatment withdrawal. Needs more social relationship support	[59]
Systemic therapy for liver mCRC	> 25	Prevention of steatosis as well as liver fibrosis is needed	[63]
Rectal cancer			
Laparoscopic surgery for rectal cancer	> 28	High difficulty of laparoscopic-assisted rectal resection	[70]
Primary full-thickness transanal excision for rectal cancer	> 27	Take steps to prevent specific surgical site complications, including anastomotic complications and pelvic abscess formation requiring surgical drainage	[71]
Patients after rectal cancer surgery	< 18.5	Poorer early and long-term prognosis	[73]
Male rectal cancer	≥ 25	Higher risk of a local recurrence	[25, 74]
Chemoradiotherapy for locally advanced rectal cancer	Loss > 7%	3-year overall survival rate significantly worse	[22]
Weight management for CRC			
Intermittent fasting	-	Only considered for use in clinical studies	[94–96]
Physical activity	-	Reduces all-cause mortality in all BMI classes, but requires attention to fatigue and cardiac function	[100, 106]
Metformin	≥ 30	Greater benefit for patients undergoing 5-FU regimen for CRC, also recommended for use in patients with other classes of BMI	[117–120, 122]
Oral nutritional supplements	-	Reduces the incidence of skeletal muscle loss and sarcopenia and improves chemotherapy tolerance in patients at nutritional risk after CRC surgery	[129]

The studies on the impact of BMI on different treatments for CRC are not comprehensive, and no studies have investigated the impact of immunotherapies such as Hartmann surgery, stenting, interventional treatments, and CAR-T. Basic research on the impact of BMI and patients with CRC is relatively sparse. Further exploration of the link between BMI and the impact of CRC treatment could also include molecular pathways and the impact of BMI on patients with CRC. In the future clinical practice, we advocate for a more comprehensive study of the interaction between BMI and cancer treatment in order to design better strategies for CRC within the PPPM paradigm.

To summarize our recommendations on the basis of our experience and reviewed knowledge we recommend:

- Following individual BMI monitoring from the pre-diagnostic period to the treatment period of CRC.
- Adopt BMI as a prognostic consideration for CRC patients in the absence of other sophisticated screening and modeling tools.
- For patients with excessively high BMI, evidence-based avoidance of risky treatment options whenever possible and planned weight loss and diet control in conjunction with treatment.
- For patients with low BMI, a combination of nutritional factors and treatment side effects should be considered.
- Appropriate physical activity for CRC patients thus helping to improve BMI and other prognostic factors. Exercise approaches should be scientifically tailored and actively recommended when the patient’s physical condition permits.
- The potential risks and benefits need to be fully explained to patients when BMI-related clinical strategies, such as intermittent fasting and metformin, for which the evidence is not yet sufficient, are used experimentally.
- Progressive BMI decline after CRC diagnosis may indicate an increased risk. It is necessary to determine the cause in time.
- BMI-based attitude within PPPM may be cost-effective and efficient, especially in developing regions.
- More research that is needed to clarify the role of BMI and potentially other indexes and factors in emerging therapies for CRC.

## Data Availability

The datasets involved in this study were extracted from publicly available papers published in the PubMed database. The datasets may be reused with the author’s permission.

## Abbreviations

*5-FU*: 5-Fluorouracil
*5FU-LEV*: 5-Fluorouracil and levamisole
*AT*: Adipose tissue
*BMI*: Body mass index
*BRS*: Bariatric surgery
*CapeOx*: Oxaliplatin plus capecitabine
*CAR-T*: Chimeric antigen receptor T-cell immunotherapy
*CI*: Confidence interval
*CR*: Chronic caloric restriction
*CRA*: Colorectal adenoma
*CRC*: Colorectal cancer
*CRS + HIPEC*: Cytoreductive surgery with hyperthermic intraoperative intraperitoneal chemotherapy
*CT*: Computed tomography
*EGFR*: Epidermal growth factor receptor
*ERAS*: Enhanced recovery after surgery
*FOLFIRI*: 5-Fluorouracil, leucovorin, and irinotecan
*FOLFOXIRI*: Folonic acid, 5-fluorouracil, oxaliplatin, and irinotecan
*HRQoL*: Health-related quality of life
*HRs*: Hazard ratios
*IF*: Intermittent fasting
*IGF*: Insulin-like growth factors
*IL*: Interleukin
*LSMI*: Linear skeletal muscle index
*mCRC*: Metastatic colorectal cancer
*MFN2*: Mitofusin-2
*mFOLFOX6*: 5-Fluorouracil, leucovorin, and oxaliplatin
*ONS*: Oral nutritional supplement
*ORs*: Odd ratios
*PA*: Physical activity
*PPPM/3PM*: Predictive, preventive, and personalized medicine
*SIR*: Standardized incidence ratio
*SMI*: Skeletal muscle index
*T2D*: Type 2 diabetes
*TNF*: Tumor necrosis factor
*TZD*: Thiazolidinediones
*VAT*: Visceral adipose tissue
*VEGF*: Vascular endothelial growth factor
*WC*: Waist circumference
*WHO*: World Health Organization
